# The South African Rugby Injury and Illness Surveillance and Prevention Project (SARIISPP): SARU Boys’ Youth Week Injury Surveillance Report, 2024

**DOI:** 10.17159/2078-516X/2026/v38i1a24858

**Published:** 2026-04-15

## Executive Summary

As part of the South African Rugby Union (SARU) Injury and Illness Surveillance and Prevention Project (SARIISPP), SARU records and investigates injury data from the annual SARU Youth Week tournaments. The BokSmart National Rugby Safety Programme has gathered and analysed these data since 2011 for the SARU Boys’ Youth Weeks.

This report focuses on the two 2024 Boys’ Rugby tournaments: the Grant Khomo Under 16 Week (GKu16) and Craven Week Under 18 (CWu18). The two tournaments had 36 participating teams, and a total of 54 matches were played. Comparisons are made between these two SARU Boys’ Youth Week tournaments in 2024 (GKu16 vs. CWu18) and between the combined 2011–2023 tournaments and the 2024 tournament. No GKu16 and CWu18 tournaments were held in 2020 and 2021 due to COVID-19 restrictions. Additionally, no data were collected at the 2022 GKu16 and CWu18 tournaments due to the continued financial impact of the COVID-19 pandemic on the programme’s resources.

Each medical facility at the SARU Youth Week tournaments has a designated researcher(s) on-site, who, together with the tournament medical doctors, records and documents the tournament injury data daily. The analysis then investigates the injury patterns for the SARU Boys’ Youth Weeks (GKu16 and CWu18). Furthermore, the analysis compares the profiles of injured players at each tournament. Throughout the analysis and while investigating these patterns, any areas of concern that may require changes to the game, tournament structure, or medical support services are identified. Consequently, injury-specific interventions can be developed and implemented when the evidence supports their need.

In 2024, a combined total of 66 time-loss injuries were recorded for both tournaments (GKu16 and CWu18). This equated to an average injury rate of 38 (29 – 47) injuries per 1000 player hours; data are expressed as mean (±95% confidence intervals) injuries per 1000 player hours. The Time-Loss injury incidence for the GKu16 and CWu18 tournaments was 42 (29 – 56) injuries per 1000 player hours and 33 (2 – 46) injuries per 1000 player hours, respectively. Analysis of the combined injury incidence data collected over the 11 included years indicated no significant difference between the two age groups. However, the CWu18 tournament tended to have a lower injury incidence.

In 2024, *Being Tackled* (ball carrier) was the most frequent injury-causing event, followed by *Open Play*, and then *Tackling* (tackler). *Being Tackled side-on (regulation)* was the most frequent injury-causing mechanism involved in Ball Carrier injuries. While *Collision in Open Play* was the most frequent injury-causing mechanism in *Open Play* injuries. Lastly, *Tackling front-on (regulation)* remained the most frequent injury-causing mechanism involved in the Tackler injuries.

The most common injury type for the combined tournaments was *Central Nervous System (CNS)* injuries. *Joint/Ligament* injuries were significantly lower than CNS injuries in the GKu16 tournament. *Head and Neck* was the most common injury location in 2024, accounting for 46% of the injuries, with 73% of these occurring in the GKu16. As expected, the injury incidence of *‘New’* injuries was higher than that of subsequent ‘*Recurrent’* injuries. Most muscle and joint/ligament injuries were ‘*New’ injuries*, and joint/ligament injuries had proportionately similar ‘*Recurrent’* injuries to muscle injuries in 2024. Players who started the match sustained more injuries than those who joined the match as substitutes. *Fullbacks* and *Wings* had the highest normalised injury incidence per player per position across the combined tournaments.

There were 26 concussions recorded in the 2024 tournaments, the same number as in 2023. Furthermore, the act of *Tackling* (the tackler) and *Open Play* each contributed to 27% of the events causing concussions. Concussion rates are similar to those in 2023.

Recommendations from this report advocate that the Boys’ rugby teams should focus on improving their tackling and ball-carrying techniques and that the coaching emphasis should increase on developing, teaching, training, and properly preparing players to execute safer contact techniques in match situations. Furthermore, strength and conditioning, along with the development of training strategies to improve peripheral vision and dynamic situational attunement and awareness during open play, are recommended to reduce open-play-related injuries.

**Figure:**
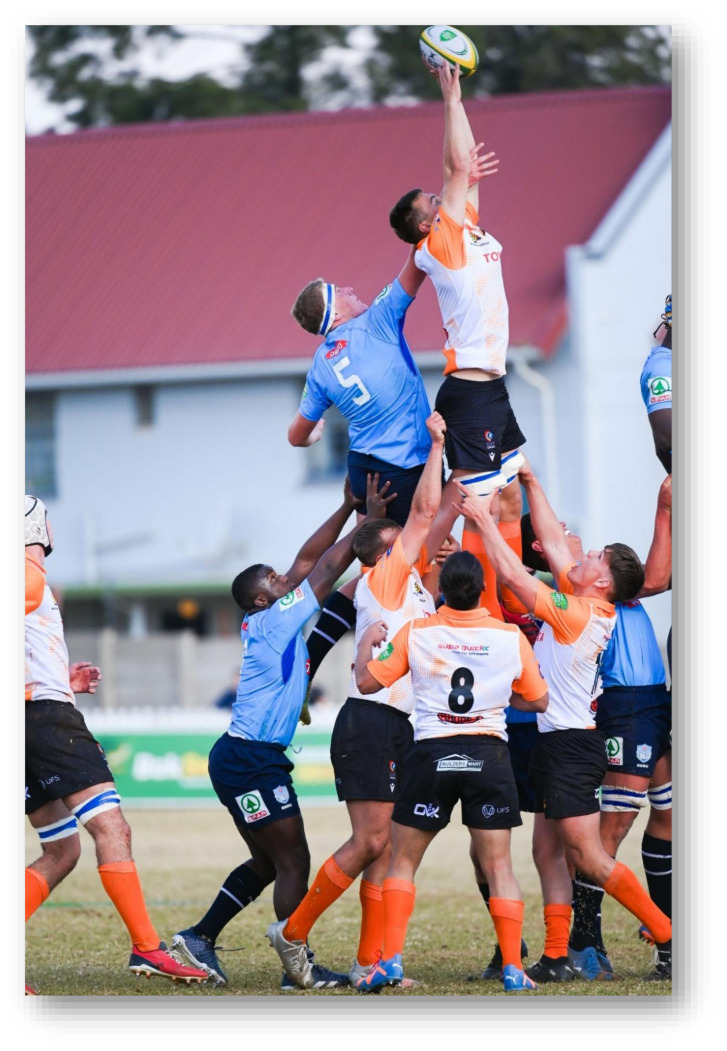


## Definitions

All definitions are based initially on the 2007 consensus statement for injury reporting in rugby union (1) and have since been realigned with the latest International Olympic Committee (IOC) consensus statement for methods of recording and reporting epidemiological data on injury and illness in sports (2).

### MEDICAL ATTENTION INJURY

All injuries seen by the tournament medical doctor or medical support staff were classified as Medical Attention injuries. These injuries are defined by the 2007 statement as an “*injury that results in a player receiving medical attention”* (1), and by the more recent IOC statement as *“a health problem that results in an athlete receiving medical attention”* (2).

### TIME-LOSS INJURY

Medical Attention injuries were further categorised as Time-Loss injuries, where appropriate, and defined by the 2007 statement as “*an injury that results in a player being unable to take a full part in future rugby training or match play*” (1). The IOC definition is *“a health problem that results in a player being unable to complete the current or future training session or competition”* (2). For clarity, this means an injury sustained by a rugby union player during a match or training session that prevented or would have prevented the player from taking full part in all rugby training activities and/or match play for more than one day following the day of injury, irrespective of whether match or training sessions were scheduled (3).

### INJURY RATE

This report defines an injury rate as the number of injuries expressed per 1000 player exposure hours. This method of expressing injury rate has been used in previous years’ Youth Week reports and other international literature, making comparisons straightforward. Moreover, the injury rate is expressed as a mean with 95% confidence intervals. A 95% confidence interval around a mean value indicates a 95% chance (i.e., very high chance) that the true value falls within this range. In this report, we present the 95% confidence intervals assuming a normal distribution of the data and use the approach of examining the overlap of the confidence intervals to determine whether the injury incidences are significantly different; if the range of confidence interval values of two comparisons does not overlap, there is a strong chance (95%) that their injury rates are different from each other. We have opted for this method because it is easy to use, conservative, and less likely to yield false-positive results (4).

### NEW, SUBSEQUENT AND RECURRENT INJURIES

A ‘*New Injury’* was defined as when a player sustained his first injury. Any injury the *same* player sustained after this initial injury was defined as a *‘Subsequent Injury’*.

According to the IOC statement, any subsequent injury to the same site and of the same type is referred to as a ‘*Recurrence’* if the index injury was fully recovered before reinjury and as an *‘Exacerbation’* if the index injury was not yet fully recovered (2).

To provide more detail on subsequent injuries for practitioners, one can further categorise the subsequent injuries into one of four groups:

- Different site - Different type
- Different site - Same type
- Same site - Different type
- Same site - Same type

According to the 2007 Consensus Statement for rugby, any subsequent injury classified as ‘Same site - Same type’ was a *‘Recurrent injury’* (1).

**Figure:**
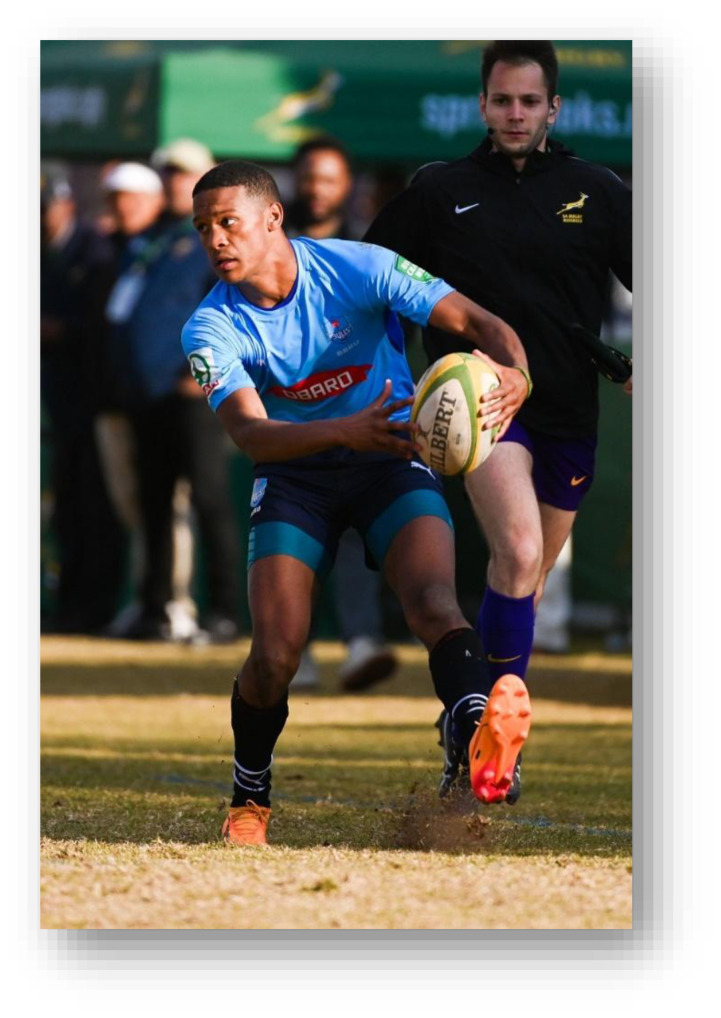


## Key Findings

### Injury Incidence

**Figure 1:**
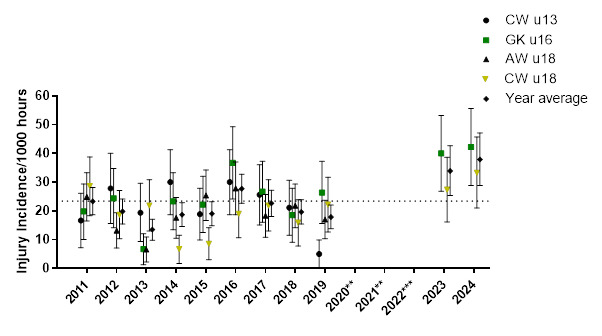
Injury incidence/1000 player hours and 95% confidence intervals of Time-Loss injuries for the SARU Boys’ Youth Week Tournaments from 2011 – 2024. The dotted line reflects the average incidence for all tournaments over all the included years. **No GKu16 and CWu18 tournaments were held in 2020 and 2021 due to COVID-19 restrictions. *** No data collection was completed in 2022 due to financial constraints.

Thirty-six teams competed in the 2024 SARU Boys’ Youth Week tournaments (GKu16 = 20 teams, CWu18 = 16 teams). A total of 54 matches were played in 2024, and 79 Medical Attention injuries were recorded during the tournaments; eighty-four percent of these (n = 66) were Time-Loss injuries. The combined tournaments’ injury incidence and 95% confidence intervals for all Medical Attention injuries were 45 (35 – 55) injuries/1000 player hours, and for Time-Loss injuries were 38 (29 – 47) injuries/1000 player hours. The incidence of Medical Attention injuries and Time-Loss injuries in the GKu16 and CWu18 were not significantly different from each other in 2024 (Table 1). The average combined injury incidence for Time-Loss injuries was higher in the 2024 SARU Boys’ Youth Week tournaments than in the other years, although this difference was not significant. The number of Medical Attention and Time-Loss injuries per match and per hour of match play across the tournaments are represented in Table 2. Figure 1 shows the pattern of Injury incidence/1000 player hours and 95% confidence intervals of Time-Loss injuries for each tournament across the years (2011 to 2024) (Figure 1).

**Figure 2:**
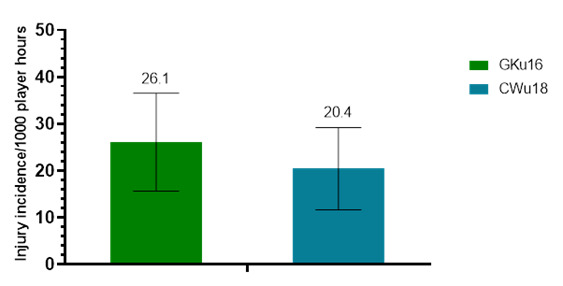
Injury incidence/1000 player hours and 95% confidence intervals at the GKu16 and CWu18 SARU Boys’ Youth Week tournaments from 2011 – 2024.

Further analysis was completed on Time-Loss injuries and for GKu16 and CWu18 tournaments only. Combined data from 2011 to 2024 shows a slightly lower, albeit not significantly different, injury incidence in CWu18, when compared to GKu16 (Figure 2).

### Injury Incidence Trends

#### U16 Grant Khomo Week (GKu16)

**Figure 3:**
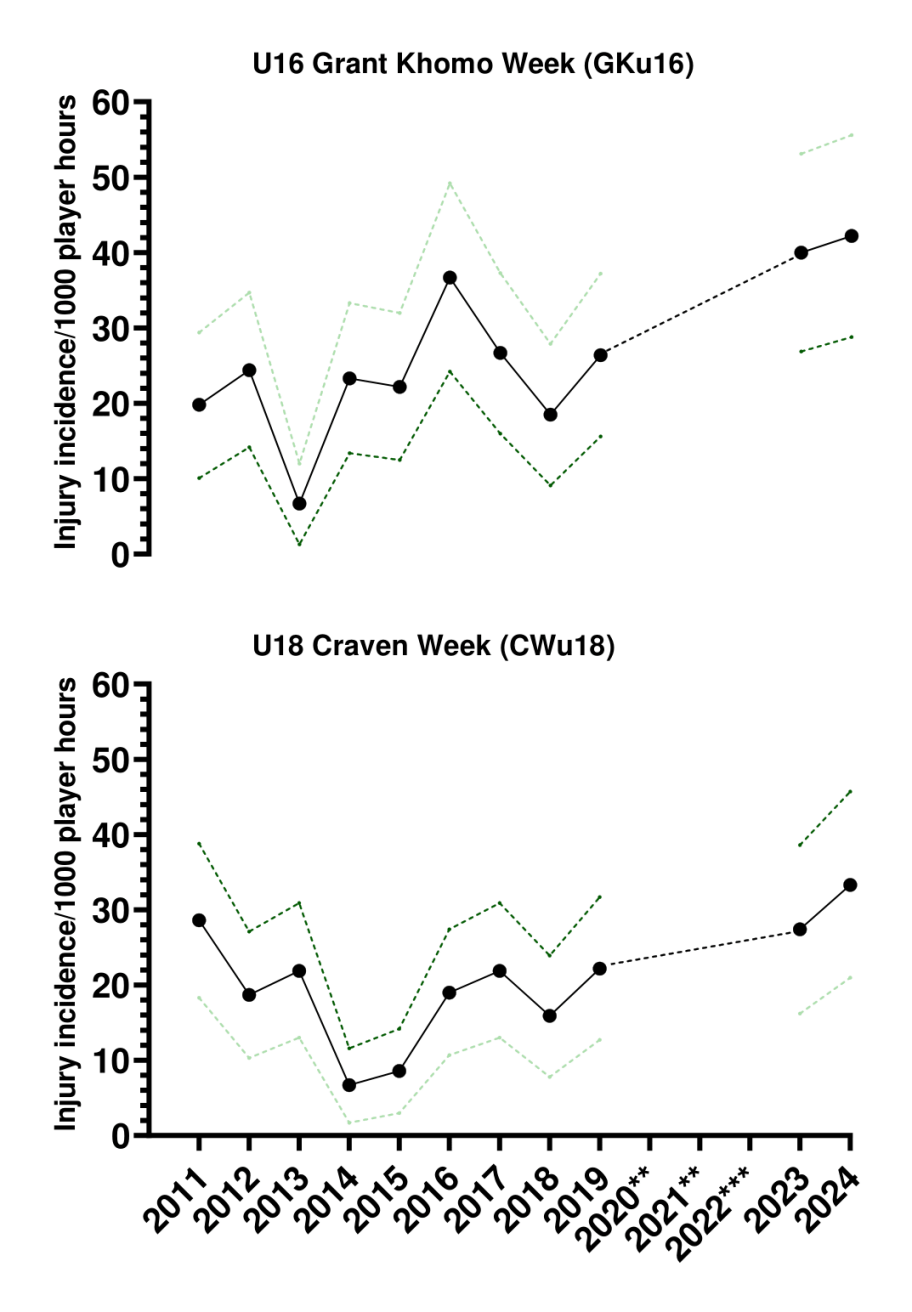
Time-Loss Injury incidence for each SARU Boys’ Youth Week tournament, per year, from 2011 – 2024, including the upper and lower 95% Confidence Intervals (95%CI). **No GKu16 and CWu18 tournaments were held in 2020 and 2021 due to COVID-19 restrictions. *** No data collection was completed in 2022 due to financial constraints.

Due to COVID-19, the GKu16 tournament was not held in 2020 and 2021, and in 2022, data collection was not conducted. Therefore, the trendline could not be calculated accurately and has been excluded. Following a decrease from 2016 to 2018, the injury incidence increased between 2018 and 2019 and again between 2019 and 2024 (Figure 3). In 2024, it was the highest recorded to date. However, the increase was not as steep as between 2019 and 2023, and in 2024, the rate was similar to that recorded in 2023.

#### U18 Craven Week (CWu18)

The CWu18 tournament was not held in 2020 and 2021 due to COVID-19, and in 2022, there was no data collection; therefore, the trendline could not be calculated accurately and was excluded. Injury incidence gradually increased each year from 2014 to 2017, then decreased in 2018. In 2019, injury incidence increased, and it increased again from 2019 to 2024 (Figure 3); 2024 was the highest injury incidence to date at this tournament.

**Figure:**
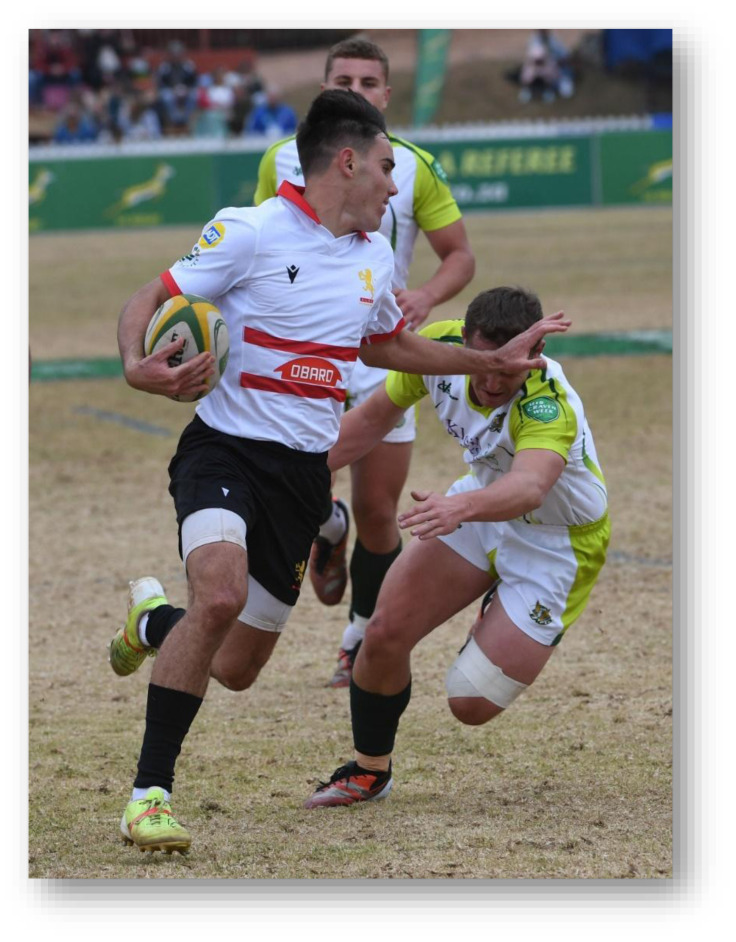


It seems as if injury rates at both tournaments have systematically increased since around 2013/2014. This could be linked to an increase in the physicality and competitiveness of the school game, with teams becoming more ‘professional’ in their coaching, player conditioning, and technical arrangements over time. It might also result from mismatches in team strength, or anecdotal feedback from medical staff at these tournaments, who notice players arriving with pre-existing niggles or injuries.

### Injury Event

Considering the combined data from the 2024 tournaments, the Ball Carrier was associated with the most injuries (33%, n = 22), followed by Open Play injuries (23%, n = 15) and Tackler-related injuries (18%, n = 12). Ball Carriers had 13 (7 – 18) injuries/1000 player hours, while Open Play-related injuries had 9 (4 – 13) injuries/1000 player hours, and Tackler injuries had 7 (3 – 11) injuries/1000 player hours.

Compared with CWu18, injury incidence among the Ball Carrier was highest in the GKu16 tournament, although the difference was not significant. Ball Carrier, Tackler and Open Play injury incidence were similar in the CWu18 tournament (Table 3). Ball Carrier injuries were higher than Tackler and Open Play in the GKu16 tournament. However, these differences were again not significant.

**Figure 4:**
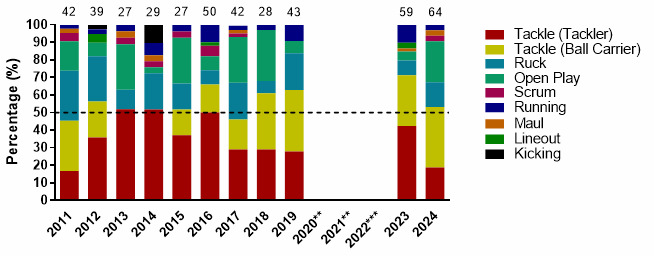
Most common injury-causing events in the GKu16 and CWu18 SARU Boys’ Youth Week tournaments from 2011 – 2024. (The number above each bar represents the total number of injuries for that year). Missing 2024 data = 2 cases. **No GKu16 and CWu18 tournaments were held in 2020 and 2021 due to COVID-19 restrictions. *** No data collection was completed in 2022 due to financial constraints.

Figure 4 displays the proportion of injuries resulting from the different injury-causing events between 2011 and 2024. There was a decrease in the proportion of injuries sustained by the Tackler from 2023 to 2024. There was an increase in the proportion of Ball Carrier injuries from 2023 to 2024. Open Play injuries increased in percentage from 2023 to 2024 (Figure 4).

**Figure 5:**
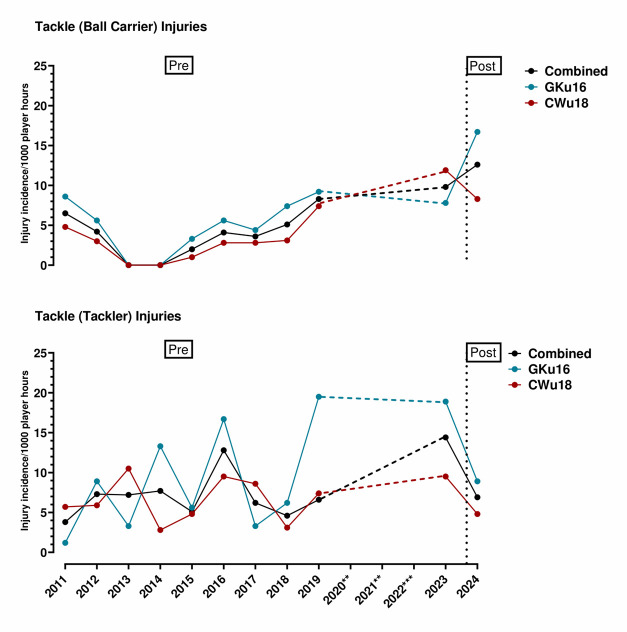
Injury incidence of Tackle (Ball Carrier) and Tackle (Tackler) injuries from 2011 to 2023 (‘pre’ lowering the legal tackle height) and 2024 (‘post’ lowering the legal tackle height) for each SARU Boys’ Youth Week tournament and the combined tournaments. **No GKu16 and CWu18 tournaments were held in 2020 and 2021 due to COVID-19 restrictions. *** No data collection was completed in 2022 due to financial constraints.

In 2024, SA Rugby lowered the legal tackle height from the shoulder level to the base of the sternum. Figure 5 displays the Tackle (Tackler) and Tackle (Ball Carrier) injuries as injury incidence per 1000 player hours from 2011 to 2023 (‘pre’ lowering the legal tackle height) and 2024 (‘post’ lowering the legal tackle height) for each SARU Boys’ Youth Week tournament and the combined tournaments. Tackler-related injuries in GKu16 fluctuated from 2011 to 2018, reaching GKu16’s highest injury incidence in 2019. Similar injury incidence was found in 2023 followed by a decrease in 2024. Tackler-related injuries in CWu18 fluctuated from 2011 to 2019. Tackler-related injuries increased again in 2023 and decreased in 2024. Tackler-related injuries in combined tournaments increased from 2011 to 2016. Tackler-related injuries proceeded to decrease to 2018. From 2019 to 2023, injuries increased again followed by a decrease in 2024. Ball Carrier-related injuries increased steadily from 2014 to 2019 in the GKu16, CWu18 and combined tournaments. From 2023 to 2024, Ball Carrier-related injuries increased in GKu16 and in the combined tournaments. In CWu18, Ball Carrier-related injuries decreased from 2023 to 2024.

**Figure:**
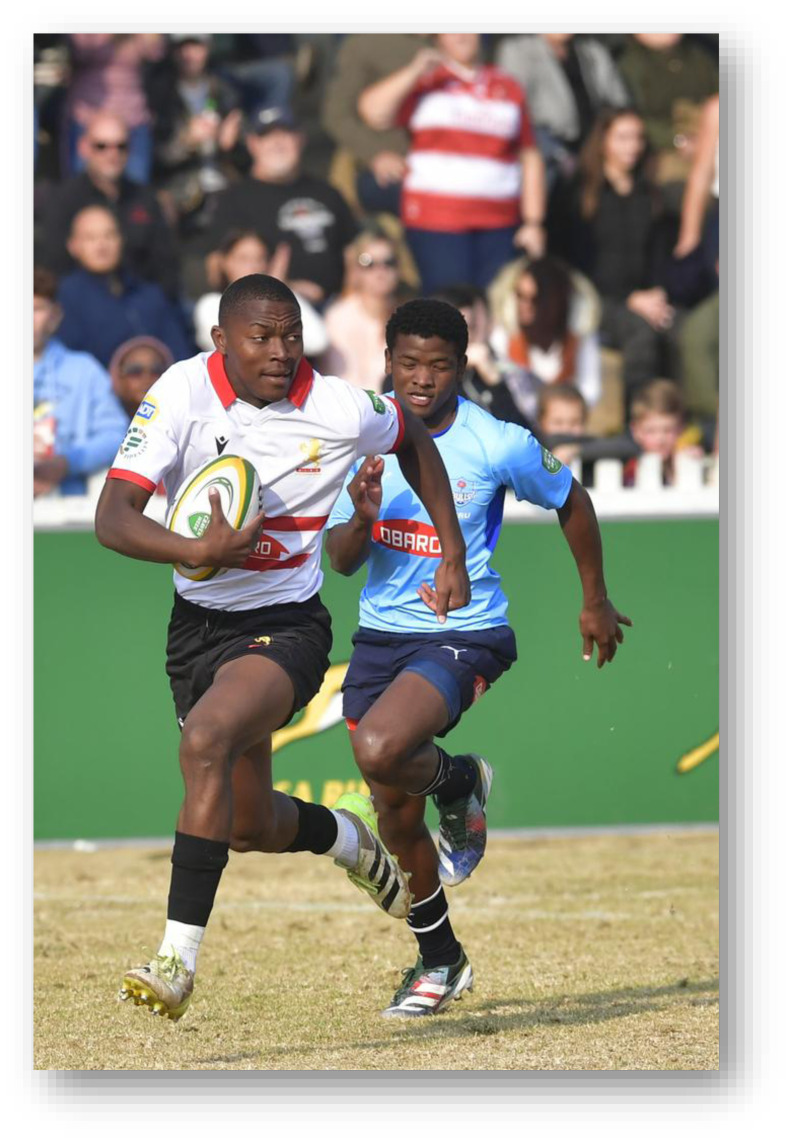


**Figure 6:**
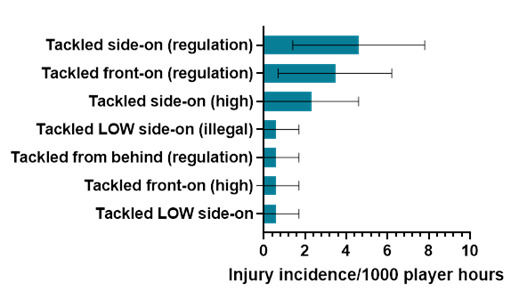
Injury incidence and 95% confidence intervals/1000 player hours of Ball Carrier-related injury mechanisms at the 2024 SARU Boys’ Youth Week Tournaments. Missing 2024 data = 0 cases.

In 2024, Tackled side-on (regulation) accounted for the highest proportion of injuries to the Ball Carrier (36%), with 4.6 (1.4 – 7.8) injuries/1000 player hours (Figure 6). This was followed by Tackled front-on (regulation) at 3.5 (0.7 – 6.2) injuries/1000 player hours.

**Figure 7:**
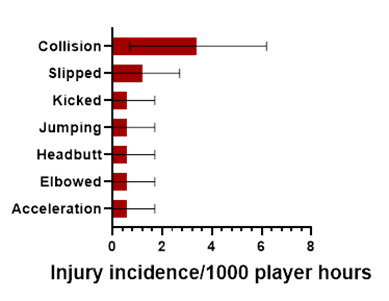
Injury incidence and 95% confidence intervals/1000 player hours of Open Play-related injury mechanisms at the 2024 SARU Boys’ Youth Week Tournaments. Missing data = 1 case.

Collision in Open Play accounted for the highest proportion of injuries in the Open Play-related injuries (50%), with 3.4 (0.7 – 6.2) injuries/1000 player hours (Figure 7).

**Figure 8:**
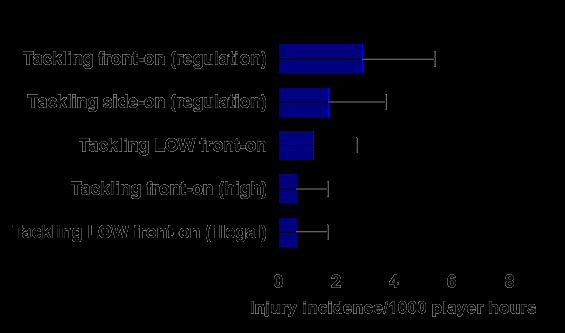
Injury incidence and 95% confidence intervals/1000 player hours of Tackler-related injury mechanisms at the 2024 SARU Boys’ Youth Week Tournaments. Missing data = 0 cases.

In 2024, Tackling front-on (regulation) accounted for the highest proportion of injuries to the Tackler (42%), with 2.9 (0.4 – 5.4) injuries/1000 player hours. Tackling side-on (regulation) accounted for 25% of injuries sustained by the Tackler, with 1.7 (0.0 – 3.7) injuries/1000 player hours (Figure 8).

**Figure:**
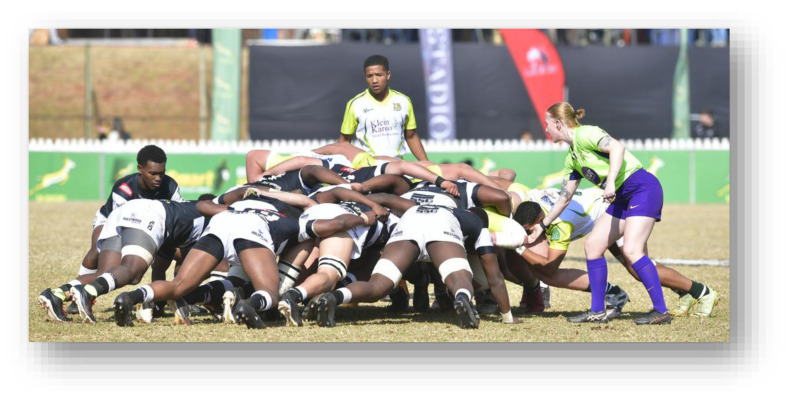


### Injury Type

In the 2024 SARU Boys’ Youth Week tournaments, the most common injury type was Central Nervous System (CNS) injuries (Table 4). Although not significant, the combined data across the tournaments showed more CNS injuries than Joint/Ligament and Muscle/Tendon injuries. The GKu16 tournament had a higher incidence of CNS and Muscle/Tendon injuries than CWu18. Joint/Ligament injury incidence was significantly lower than CNS injuries in the GKu16 tournament, yet it was the greatest contributor to injuries in the CWu18.

**Figure 9:**
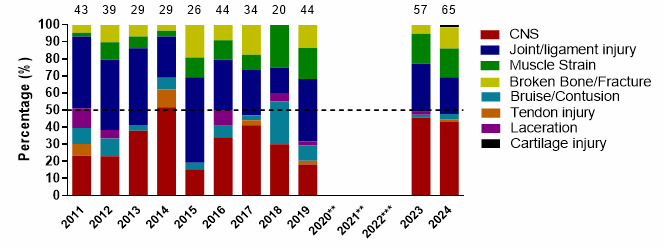
Most common injury types in the GKu16 and CWu18 SARU Boys’ Youth Week tournaments from 2011 – 2024. (The number above each bar represents the total number of injuries for that year). Missing 2024 data = 1 case. **No GKu16 and CWu18 tournaments were held in 2020 and 2021 due to COVID-19 restrictions. *** No data collection was completed in 2022 due to financial constraints.

Figure 9 illustrates the proportional contribution of the most common injury types per year from 2011 to 2024. CNS injuries were similar in 2023 and 2024. Joint/Ligament-related injuries decreased slightly in 2024, and Broken Bone/Fractures increased noticeably.

### Body Location

All injuries from the 2024 tournaments were grouped according to the four main body locations (*Head & Neck; Trunk; Upper Body; Lower Body*). In the 2024 tournaments, the most common injured body location was the *Head & Neck* (46%), with 73% of these occurring at the GKu16 tournament. *Lower Body* injuries were the second most common injured body location. *Lower Body* recorded an injury incidence of 15 (9 – 21) injuries/1000 player hours (Table 5), while the *Upper Body* injury incidence was 5 (1 – 8) injuries/1000 player hours. Both *Trunk* and *Upper Body* injury incidence were significantly lower than both *Head & Neck* and *Lower Body* locations.

Table 6 presents the 2024 SARU Boys’ Youth Week Tissue and Pathology injury data in the format recommended by the IOC consensus statement (2).

### New vs. Recurrent

The injury incidence of *‘New’* injuries in 2024 was 33 (24 – 41) injuries/1000 player hours; a slightly higher injury incidence than in 2023. The subsequent ‘*Recurrent’* injuries were 5 (2 – 9) injuries/1000 player hours, which was lower than in 2023.

Most muscle injuries (73%) and joint/ligament injuries (71%) were *‘New’* injuries, and joint/ligament injuries had a similar percentage of *‘Recurrent’* injuries (29%) to muscle injuries (27%) in 2024.

**Figure 10:**
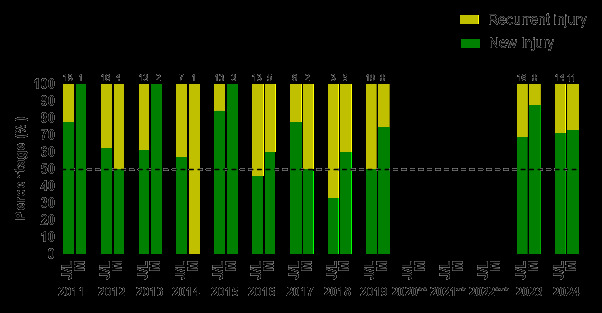
Proportion of New and Recurrent joint/ligament and muscle injuries in the GKu16 and CWu18 SARU Boys’ Youth Week tournaments from 2011 – 2024. (The number above each bar represents the total number of injuries for that year). J/L = Joint/Ligament and M = Muscle. **No GKu16 and CWu18 tournaments were held in 2020 and 2021 due to COVID-19 restrictions. *** No data collection was completed in 2022 due to financial constraints.

Figure 10 illustrates the proportion of *‘New’* and *‘Recurrent’* joint/ligament, and muscle injuries across the years (2011–2024). There was a similar proportion of *‘Recurrent’* joint/ligament injuries from 2023 (31%) to 2024 (29%), and *‘Recurrent’* muscle injuries increased from 2023 (13%) to 2024 (27%).

There was a slight reciprocal increase in the proportion of *‘New’* joint/ligament injuries from 69% in 2023, to 71%, in 2024. The proportion of *‘New’* muscle injuries decreased from 88% in 2023 to 73% in 2024.

### Game Quarter

**Figure 11:**
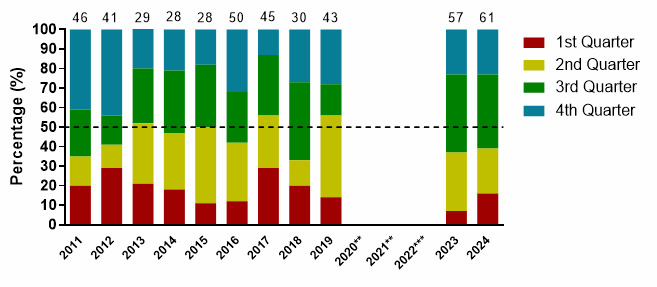
Proportion of injuries occurring in each game quarter in the GKu16 and CWu18 SARU Boys’ Youth Week tournaments from 2011 – 2024. (The number above each bar represents the total number of injuries for that year). Missing 2024 data = 5 cases. **No GKu16 and CWu18 tournaments were held in 2020 and 2021 due to COVID-19 restrictions. *** No data collection was completed in 2022 due to financial constraints.

Injuries for the 2024 tournaments mainly occurred in the 3^rd^ quarter (35%); 13 (8 – 19) injuries/1000 player hours. First (1^st^) quarter injuries increased from 2023 to 2024, while second (2^nd^) quarter injuries decreased (Figure 11).

**Figure:**
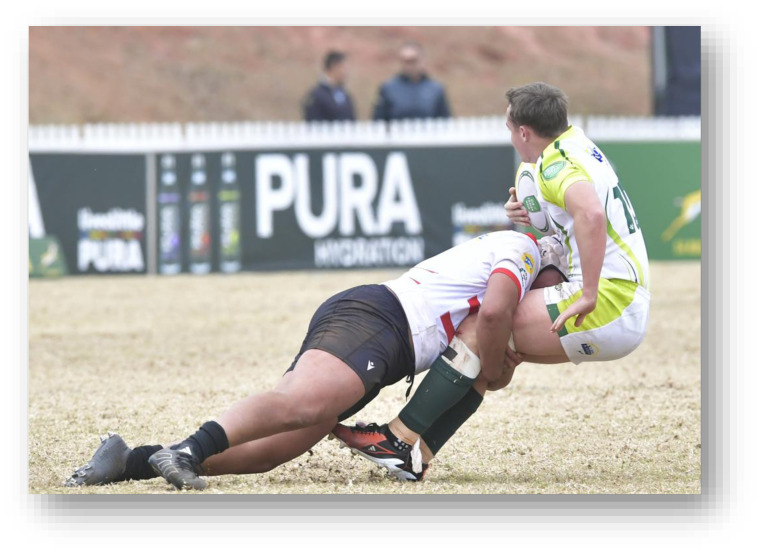


### Match status

**Figure 12:**
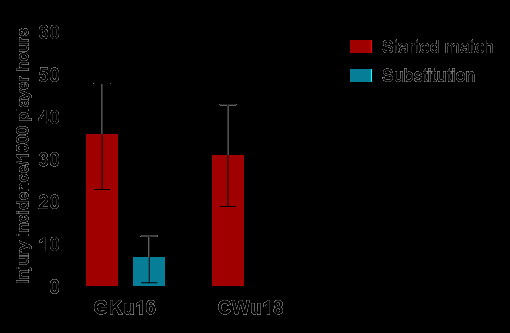
Injury incidence and 95% confidence intervals/1000 exposure hours for players who started the match and those who came on as substitutes, in the 2024 SARU Boys’ Youth Week Tournaments.

In 2024, there were significantly more injuries among players who started the match (91%) than among players who entered as substitutes (9%) (Figure 12). There were no significant differences in injury rates between tournaments for starters. There were no injuries for players who came on as substitutes in the CWu18 tournament (Table 7).

### Player positions

**Figure 13:**
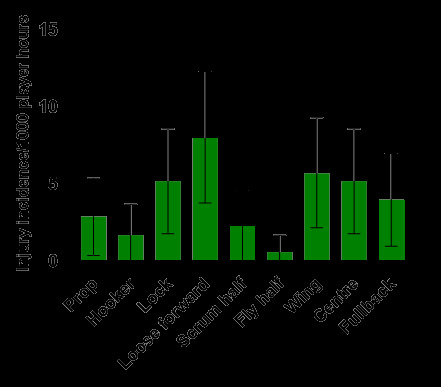
Absolute injury incidence and 95% confidence intervals/1000 player hours per position in the SARU Boys’ Youth Week Tournaments 2024.

In 2024, the loose forward and wing positions had the highest *absolute* injury incidence rates across the SARU Boys’ Youth Week tournaments (Figure 13). Loose forwards and wings had an *absolute* injury incidence of 8.0 (3.8 – 12.3) injuries/1000 player hours and 5.7 (2.2 – 9.3) injuries/1000 player hours, respectively. *Absolute* incidence refers to the incidence of injury in a player’s positional grouping, e.g., wings, without normalising for the number of players on the field playing in that positional grouping, e.g., there are two wings *per team* on the field.

The number of injuries was also *normalised* to the number of players on the field per team, within each positional grouping. For example, Props = total number of injuries divided by 2, Locks = total number of injuries divided by 2, and Loose forwards = total number of injuries divided by 3.

**Figure 14:**
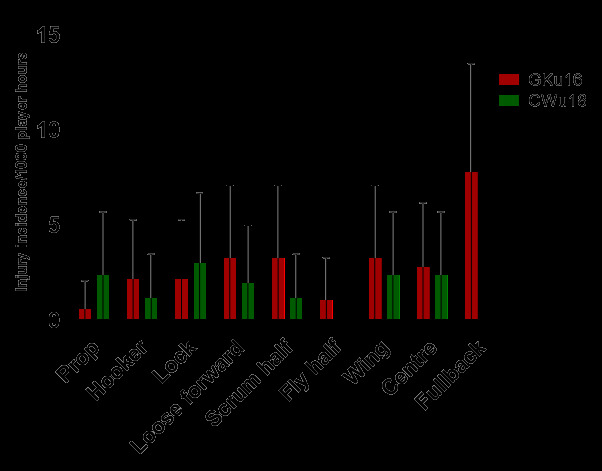
Normalised injury incidence and 95% confidence intervals/1000 player hours per player, per position, per team, across the two SARU Boys’ Youth Week Tournaments in 2024.

**Figure 15:**
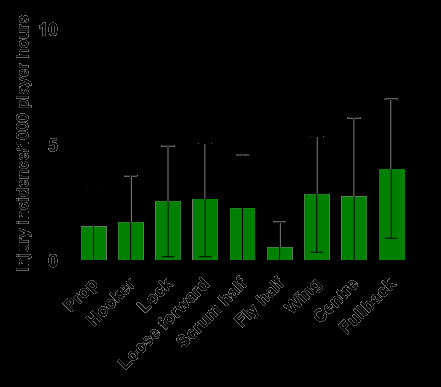
Normalised injury incidence and 95% confidence intervals/1000 player hours per player, per position, per team, from the combined SARU Boys’ Youth Week Tournaments in 2024.

Figure 14 shows the *normalised* injury incidence per player per position across the two tournaments. In the GKu16, Fullbacks stood out, and in the CWu18, Locks stood out. Figure 15 shows the combined *normalised* positional injury rates from both tournaments. In 2024, the fullback and wing positions had the highest *normalised* injury incidence rates when combining the data. Fullbacks, when normalised per player, had an injury incidence of 4.0 (1.0 – 7.0) injuries/1000 player hours and Wings had an injury incidence of 2.9 (0.4 – 5.4)/1000 player hours (Figure 15).

**Figure:**
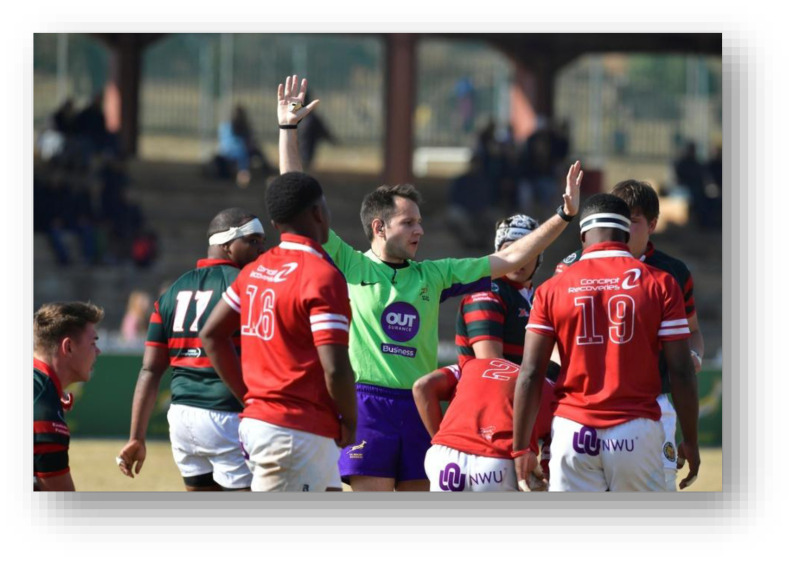


**Figure:**
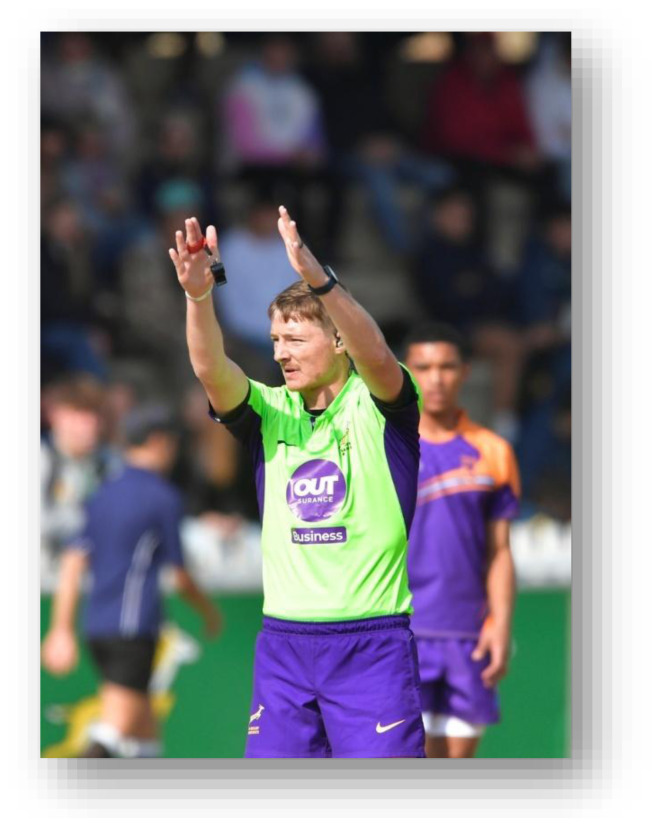


### Concussion

There was a total of 26 concussions recorded over the two tournaments played in 2024, resulting in an incidence rate of 15 (9 – 21) concussions/1000 player hours and approximately one concussion for every two matches played.

The GKu16 was the tournament with the highest concussion incidence rate of 20 (11 – 29) concussions/1000 player hours (Table 8). These data converted to 2 matches/concussion event. Albeit that most of the concussions occurred in the GKu16 (18 of the 26), the two tournaments’ concussion rates were not significantly different from each other.

**Figure 16:**
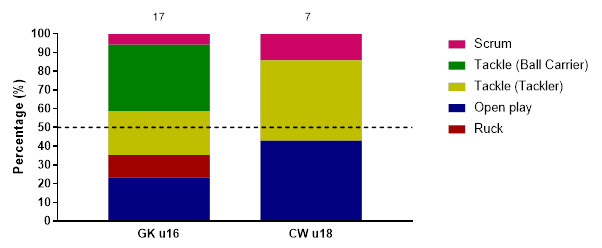
Proportion of concussions caused by the different injury events at the 2024 SARU Boys’ Youth Week Tournaments (n = 26 concussions, missing cases = 2).

**Figure 17:**
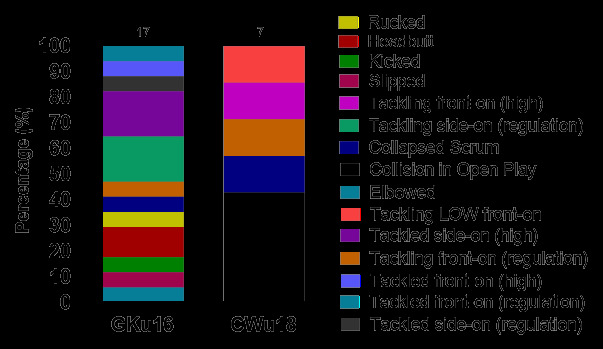
Proportion of concussions caused by the different injury event-mechanisms at the 2024 SARU Boys’ Youth Week Tournaments. Missing cases = 2 (The number above each bar represents the total number of concussions for that Tournament).

In 2024, Tackling (27%, n = 7), Open Play (27%, n = 7), and being Tackled (23%, n = 6) were the three events that contributed to the most concussions (Figure 16). Figure 17 displays the proportion of concussions caused by the different injury event mechanisms across the tournaments in 2024. Although the numbers are too low to make any firm conclusions, tackling side-on (regulation) and tackled side-on (high) contributed to the highest proportion of concussions in GKu16 (18% each) and Collision in Open Play to the highest proportion of concussions in CWu18 (43%).

**Figure 18:**
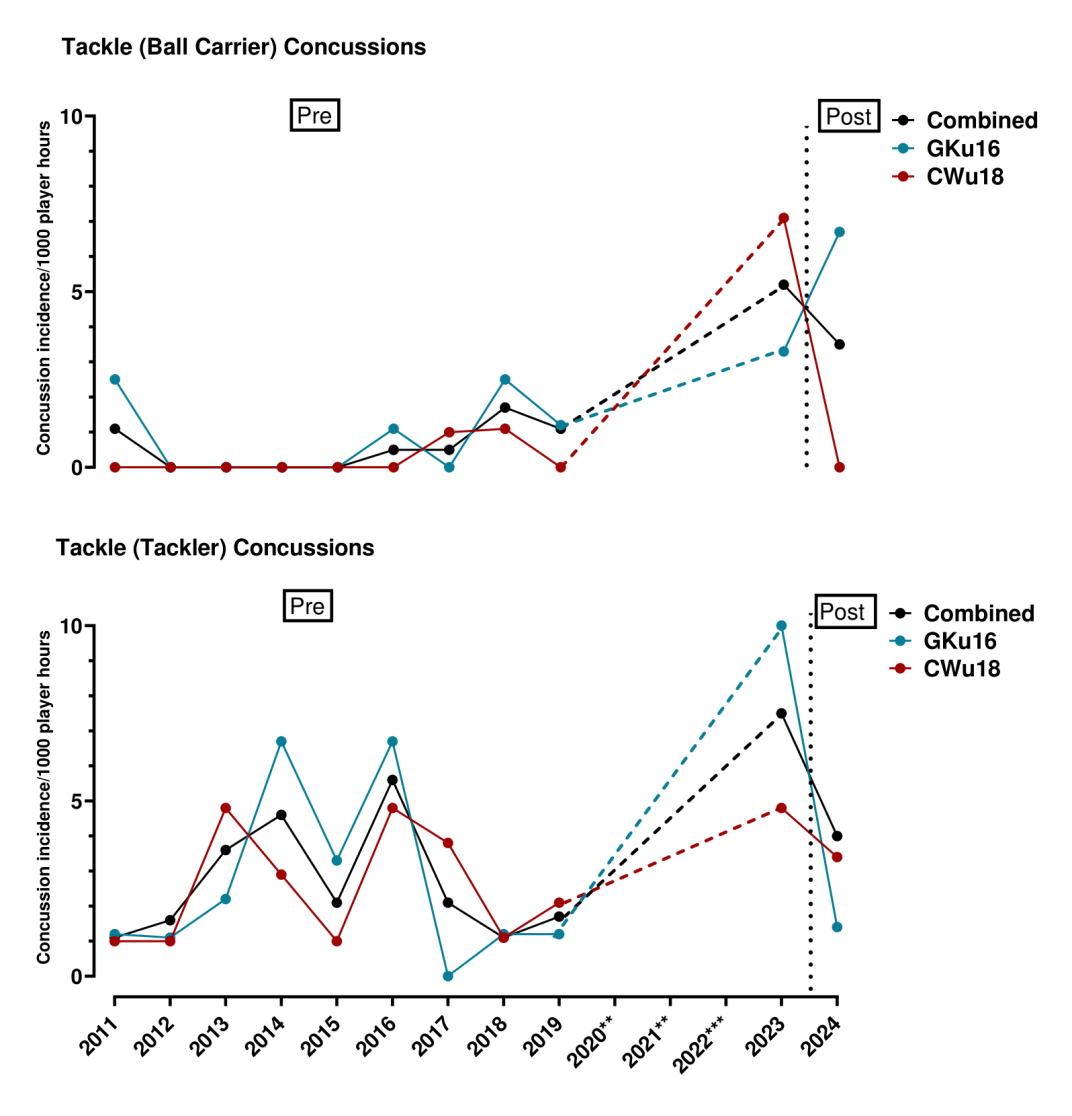
Incidence of Tackle (Ball Carrier) and Tackle (Tackler) concussions from 2011 to 2023 (‘pre’ lowering the legal tackle height) and 2024 (‘post’ lowering the legal tackle height) for each SARU Boys’ Youth Week tournament and the combined tournaments. **No GKu16 and CWu18 tournaments were held in 2020 and 2021 due to COVID-19 restrictions. *** No data collection was completed in 2022 due to financial constraints.

Figure 18 displays the Tackle (Tackler) and Tackle (Ball Carrier) injuries as concussion incidence per 1000 player hours from 2011 to 2023 (‘pre’ lowering the legal tackle height) and 2024 (‘post’ lowering the legal tackle height) for each SARU Boys’ Youth Week tournament and the combined tournaments. Tackler-related concussions in GKu16 fluctuated from 2011 to 2016. In 2017, Tackler-related concussions decreased to the lowest concussion rate for GKu16. Tackler-related concussions increased from 2017 to 2023. Tackler concussion incidence decreased in 2024 to a rate similar to that recorded in 2019. Tackler-related concussions in CWu18 fluctuated from 2011 to 2018. Tackler-related concussions increased from 2018 to 2023. In 2024, Tackler-related concussions decreased. Tackler-related concussions in the combined tournaments fluctuated from 2011 to 2018. The combined incidence of these injuries increased in 2023. In 2024, Tackler-related injury incidence decreased again. Ball Carrier-related concussions remained low from 2011 to 2015 in the GKu16, CWu18 and combined tournaments. Ball Carrier-related concussions fluctuated from 2015 to 2019. The incidence then increased from 2019 to 2023. Ball Carrier-related concussions in CWu18 and combined tournaments decreased from 2023 to 2024. In GKu16, Ball Carrier-related concussions continued to increase.

**Figure 19:**
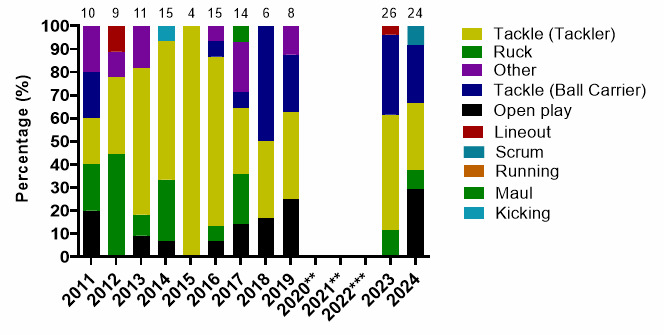
Proportion of concussions caused by the different injury events from 2011 to 2024 GKu16 and CWu18 SARU Boys’ Youth Week Tournaments (The number above each bar represents the total number of concussions for that year). Missing 2024 cases = 2. **No GKu16 and CWu18 tournaments were held in 2020 and 2021 due to COVID-19 restrictions. *** No data collection was completed in 2022 due to financial constraints.

In Figure 19, the number of all concussions in 2024 was similar to 2023. The number of Tackler- and Ball Carrier-related concussions, however, decreased in 2024, in which the lowered tackle-height laws were implemented for the first time, while the amount of Open Play injury mechanisms causing concussions increased substantially.

**Figure 20:**
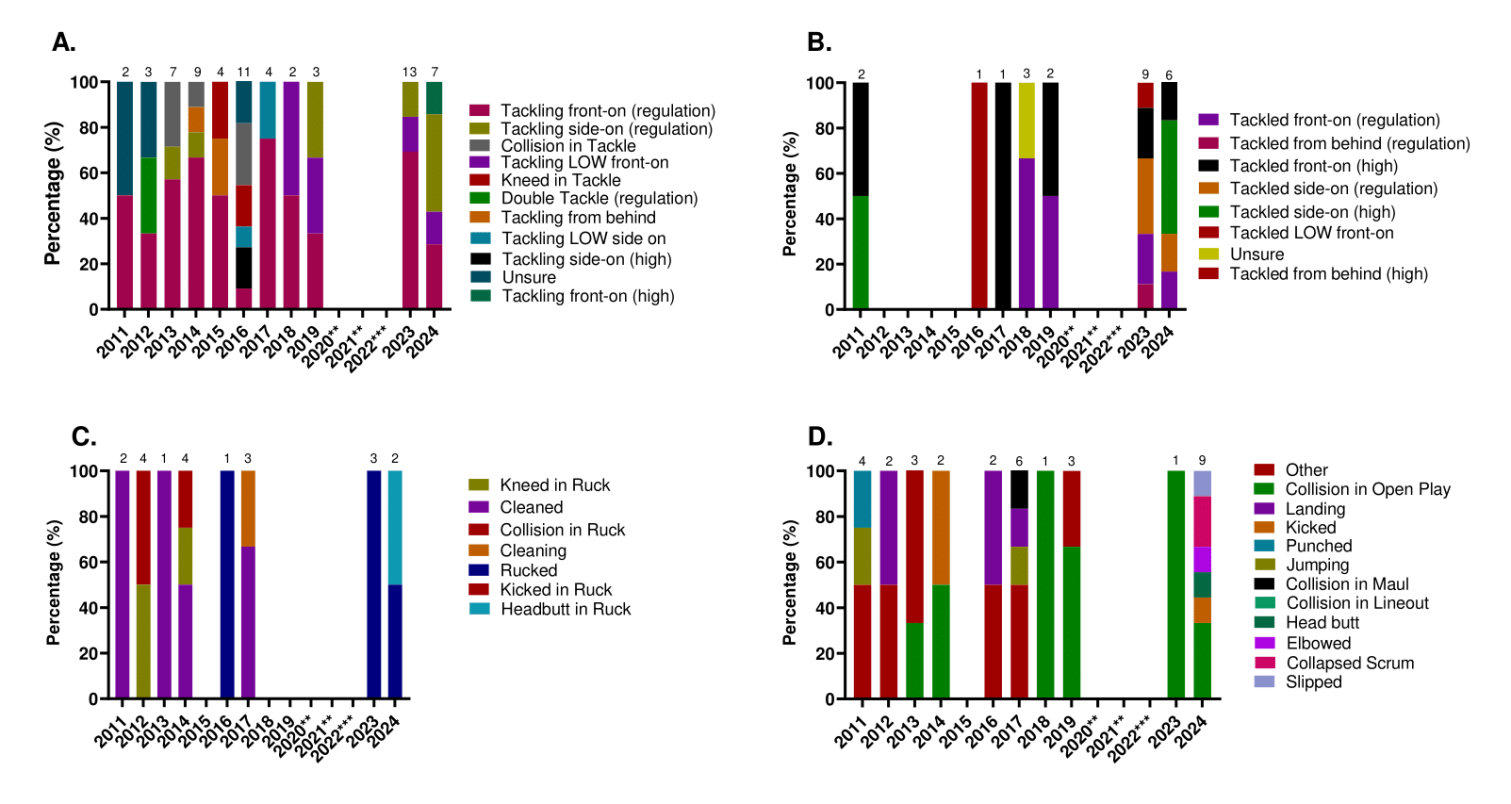
Proportionate breakdown of concussions caused by the various injury event-mechanisms at the 2011 to 2024 GKu16 and CWu18 SARU Boys’ Youth Week Tournaments. (The number above each bar represents the total number of concussions for that year). A – Tackler-related concussion mechanisms. B – Ball Carrier-related concussion mechanisms. C – Ruck-related concussion mechanisms. D – Remaining concussion mechanisms. Missing 2024 cases = 2 cases. **No GKu16 and CWu18 tournaments were held in 2020 and 2021 due to COVID-19 restrictions. ***No data collection was completed in 2022 due to financial constraints.

Figure 20 shows the percentage of concussions from various event-mechanisms over the eleven years studied. Between 2011 and 2024, 48% of Tackler-related concussions (Figure 20A) were caused by *tackling front-on (regulation)*, 25% of Ball Carrier-related concussions (Figure 20B) were caused by being *tackled front-on (regulation)* and *tackled front-on (high)*, and 35% of Ruck-related concussions were caused by being *Cleaned in the Ruck* (Figure 20C). Of the remaining concussion mechanisms (Figure 20D), *Collisions in Open Play* contributed the most, at 24%.

**Figure 21:**
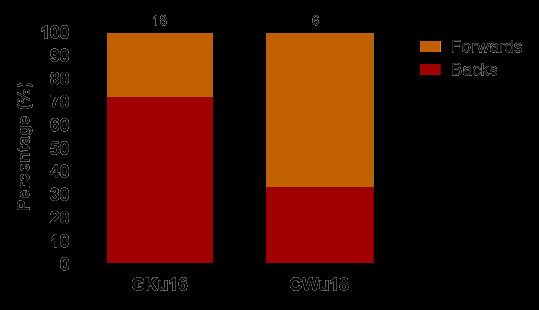
Proportionate breakdown of concussions for forwards and backs at the 2024 SARU Boys’ Youth Week Tournaments. Missing cases = 2 (The number above each bar represents the total number of concussions for that tournament).

Most of the concussions in 2024 occurred to backs (58%), and mainly in GKu16 (Figure 21).

**Figure 22:**
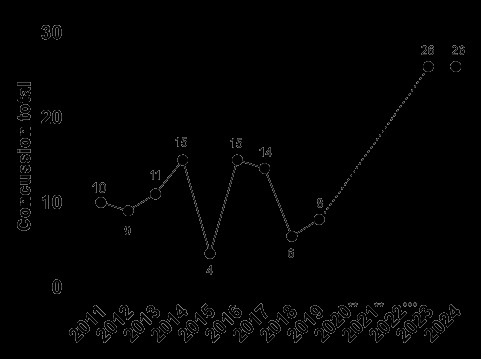
Total number of concussions per year at the GKu16 and CWu18 SARU Boys’ Youth Week Tournaments from 2011 – 2024. (The number above each data point represents the total number of concussions for that year). **No GKu16 and CWu18 tournaments were held in 2020 and 2021 due to COVID-19 restrictions. *** No data collection was completed in 2022 due to financial constraints.

**Figure 23:**
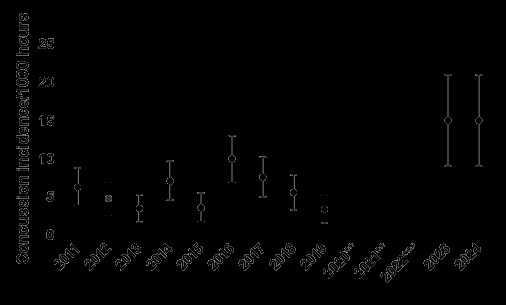
Concussion incidence rates and 95% confidence intervals/1000 player hours per year at the GKu16 and CWu18 SARU Boys’ Youth Week Tournaments from 2011 – 2024. **No GKu16 and CWu18 tournaments were held in 2020 and 2021 due to COVID-19 restrictions. *** No data collection was completed in 2022 due to financial constraints.

From 2011 to 2024, the total number and rate of concussions followed a specific pattern. Both the total number (Figure 22) and rate (Figure 23) for GKu16 and CWu18 initially increased until 2014, then sharply decreased in 2015. In 2016, both the number and the rate were similar to those in 2013–2015, and then gradually decreased until 2018. However, the concussion rate increased substantially in 2023, exceeding expectations. This significant 2023 increase may be attributed to a combination of heightened awareness of concussions and the disruption in rugby player development and participation in 2020 and 2021 due to COVID-19. In 2024, however, the concussion total and rate remained higher, like in 2023 (Figures 22 and 23).

**Figure:**
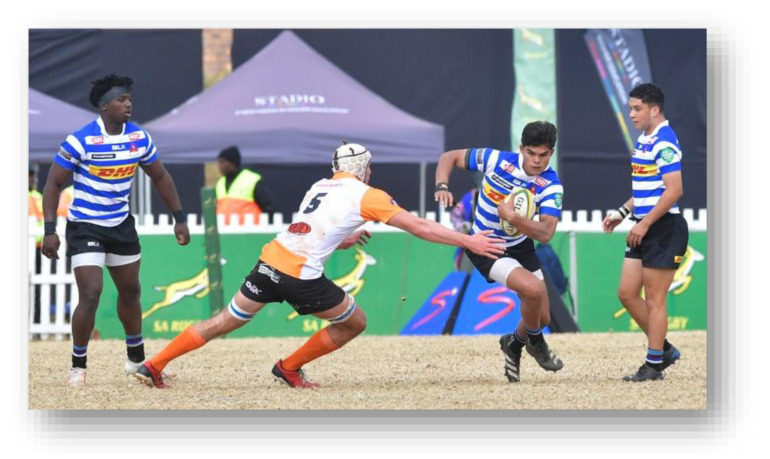


**Figure:**
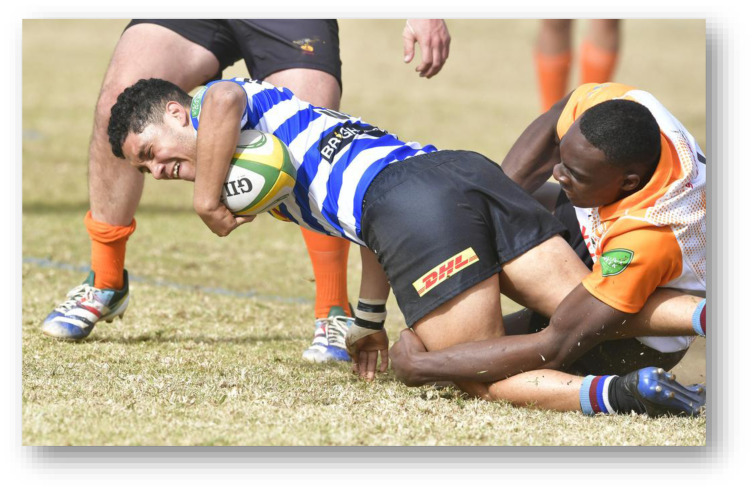


**Figure 24:**
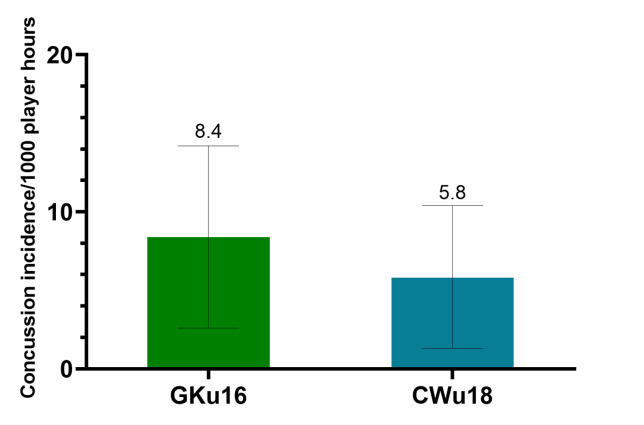
Grouped concussion incidence rates and 95% confidence intervals/1000 player hours per SARU Boys’ Youth Week tournament from 2011 – 2024.

The grouped average concussion incidence (2011 – 2024), even though not significantly different, decreases as the age and level of play increases (Figure 24).

**Figure:**
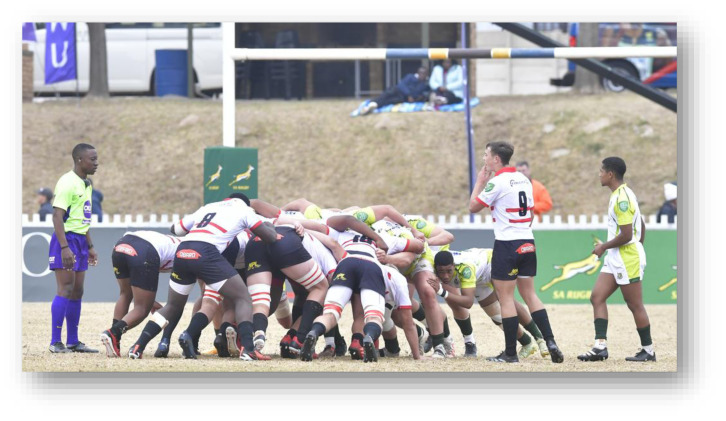


**Figure 25:**
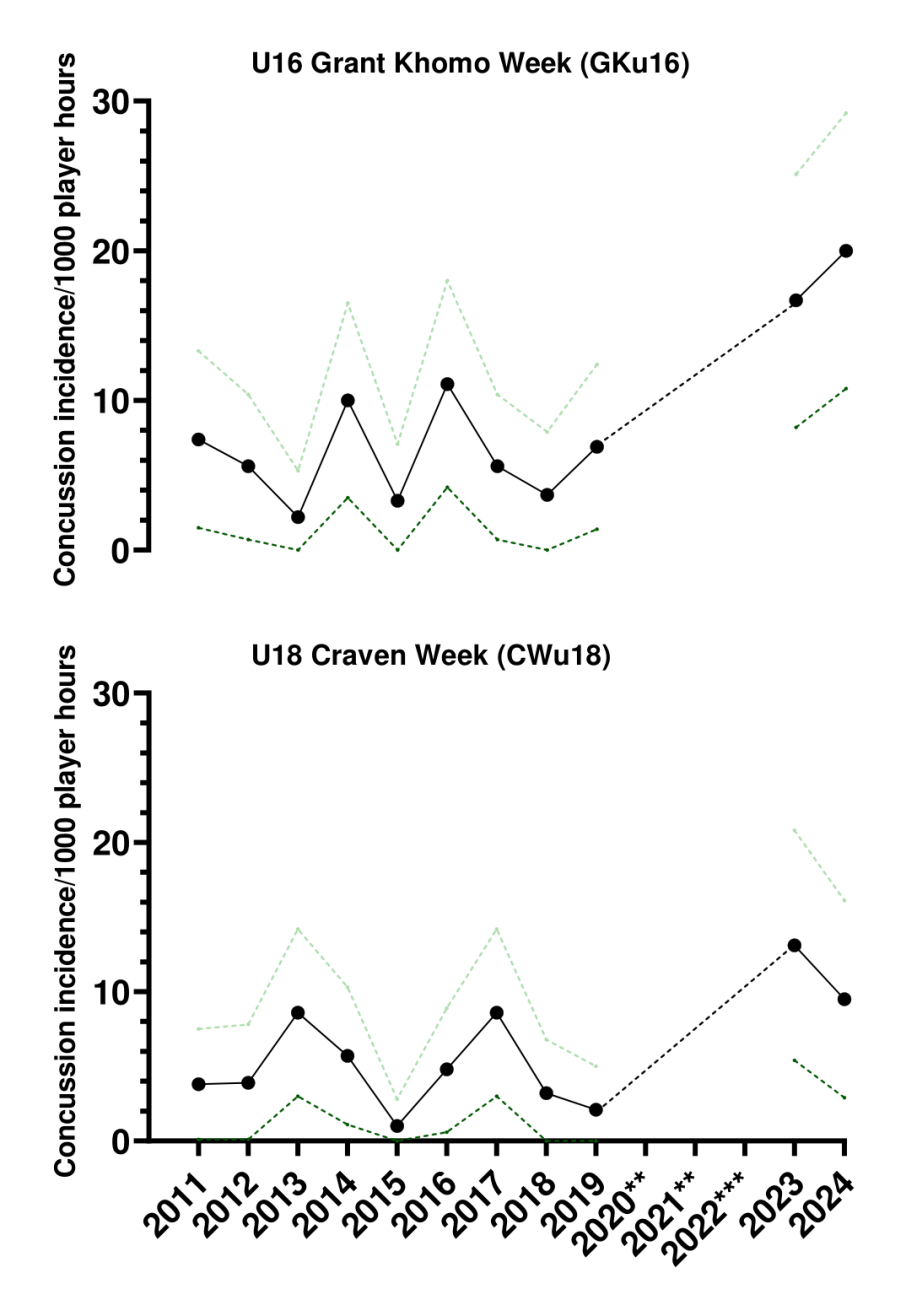
Concussion incidence and 95%CI for each SARU Boys’ Youth Week tournament, from 2011 – 2024. **No GKu16 and CWu18 tournaments were held in 2020 and 2021 due to COVID-19 restrictions. *** No data collection was completed in 2022 due to financial constraints.

The incidence of concussions varied across individual tournaments over time. It initially increased from around 2013 due to improved concussion education, awareness, and stricter protocols, but remained relatively low after 2017 (see Figure 25) until the COVID-19 pandemic. Concussions among GKu16 players increased significantly from 2019 to 2024, while CWu18 players experienced a decrease from 2017 to 2019 and then increased in 2023. In 2024, concussions decreased slightly in the CWu18 but remain elevated compared to previous levels before COVID-19.

**Figure:**
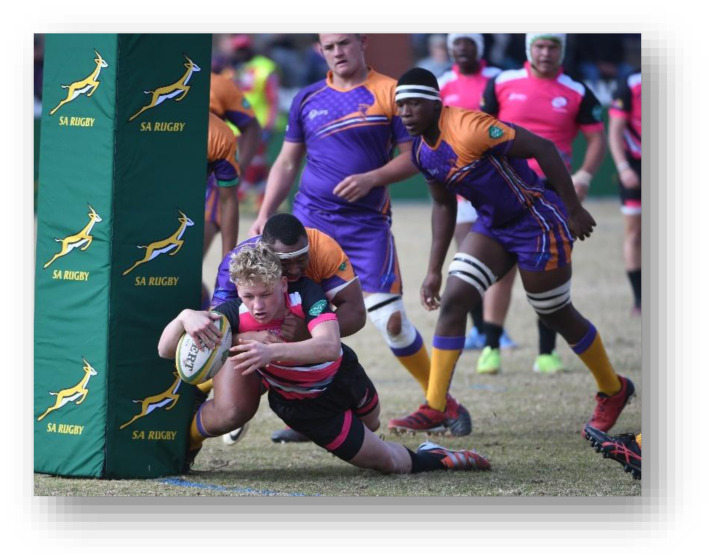


## Take-home message

1. Injuries at both tournaments have systematically increased since around 2013/2014. This could be related to a corresponding increase in the physicality and competitiveness of the school game and school teams becoming more ‘professional’ in their coaching, player conditioning, and technical setups over time. It could also be because of team strength versus strength mismatches, or anecdotally from the medical staff working at these tournaments, due to players arriving at the tournaments with existing injuries. We acknowledge this potential trend in injury rates at this level and will monitor it to ensure that it does not exceed acceptable or comparable limits.
2. The tackle and open play events caused most of the time-loss injuries and concussions, with ball carrier injuries being more prolific.
3. Since most injuries to the tackler and ball carrier occurred during regulation tackles, it remains essential to focus on developing, teaching, and practising correct, safe, and effective tackle and ball-carrying techniques to minimise tackle-related injuries.
4. The number and rate of concussions remain elevated after the 2023 increase.
5. The number of tackler- and ball carrier-related concussions decreased in 2024. This decline in tackle-related concussions may be linked to the lowering of the legal tackle height. However, with this SARU law change, contact technique and correct body positioning have become more important for continued injury prevention.
6. Strengthening and developing training strategies to enhance peripheral vision and dynamic situational awareness during open play are recommended to reduce collision injuries related to open play. These will also help mitigate ball carrier injuries.

**Figure:**
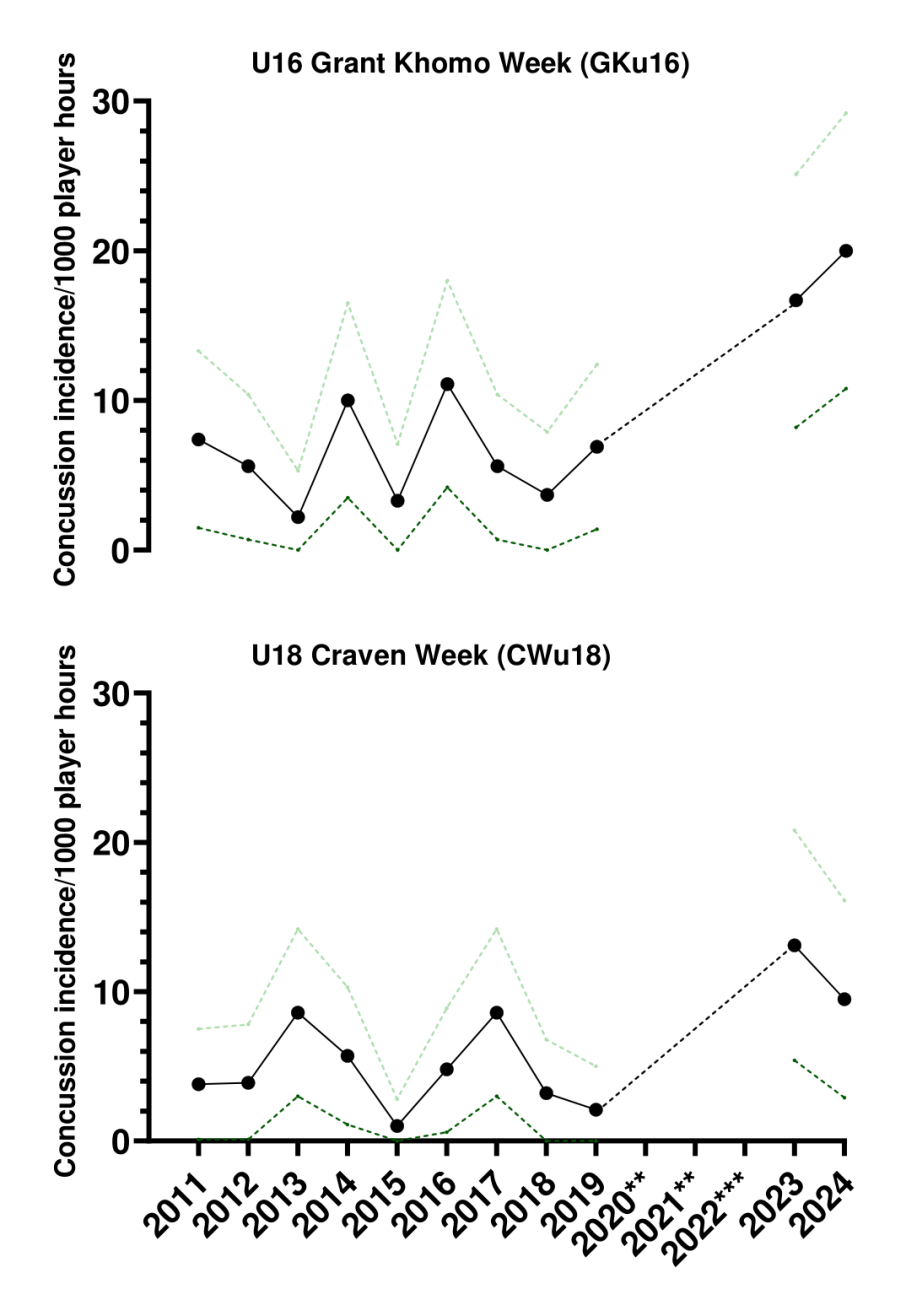


**Table 1:** Number and injury incidence (95% CI)/1000 player hours of Medical Attention and Time-Loss injuries in the 2024 SARU Boys’ Youth Week tournaments.

	Medical Attention Injuries		Time-Loss Injuries	

	Number	Incidence	Number	Incidence
**GKu16**	44	49 (34 – 63)	38	42 (29 – 56)
**CWu18**	35	42 (28 – 56)	28	33 (21 – 46)

***Combined Total***	***79***	***45 (35 –55)***	***66***	***38 (29 – 47)***

**Table 2:** Number of Medical Attention and Time-Loss injuries. Data expressed per match and per hour played in the 2024 SARU Boys’ Youth Week tournaments.

Tournament	Number of matches	Match duration (mins)	Medical Attention injuries/match	Time-Loss injuries/match	Medical Attention injuries/hour	Time-Loss injuries/hour
**GKu16**	30	60	1.5	1.3	1.5	1.3
**CWu18**	24	70	1.5	1.2	1.3	1.0

***Combined Tournament Average***	***27***	***65***	***1.5***	***1.2***	***1.4***	***1.1***

**Table 3:** Injury incidence (95% CI)/1000 player hours of Time-Loss injuries to the Tackler roles, Ball Carrier roles, and Open Play injuries for the 2024 SARU Boys’ Youth Week tournaments.

Tournament	Ball Carrier	Open Play	Tackler
**GKu16**	17 (8 – 25)	8 (2 – 14)	9 (3 – 15)
**CWu18**	8 (2 – 15)	10 (3 – 16)	5 (1 – 9)

***Combined total***	***13 (7 – 18)***	***9 (4 – 13)***	***7 (3 – 11)***

**Table 4:** Injury incidence (95% CI)/1000 player hours of Time-Loss injuries at the 2024 SARU Boys’ Youth Week tournaments grouped as Central Nervous System (CNS), Joint/Ligament, and Muscle/Tendon injuries.

Tournament	CNS	Joint/Ligament	Muscle/Tendon
**GKu16**	22 (13 – 32)	4 (1 – 9) *	9 (3 – 15)
**CWu18**	10 (3 – 16)	12 (5 – 19)	5 (0 – 9)

***Combined Total***	***16 (10 – 22)***	***8 (4 – 12)***	***7 (3 – 11)***

* GKu16 Joint/Ligament injuries were significantly lower than GKu16 CNS injuries.

**Table 5:** Proportion (%) and incidence (95% CI)/1000 player hours of Time-Loss injuries, grouped by body location, in the 2024 SARU Boys’ Youth Week tournaments.

	Proportion of injuries (%)	Incidence (95% CI)/1000 player hours
**Head & Neck**	46	17 (11–23)
**Lower Body**	39	15 (9–21)
**Upper Body**	12	5 (1–8) *
**Trunk**	3	1 (0–3) *

* Significantly lower than *Head & Neck* and *Lower Body* grouped Locations

**Table 6:** Injuries grouped according to the IOC recommended categories of Tissue and Pathology types for the 2024 SARU Boys’ Youth Week tournaments.

Tissue	Incidence	Mean time loss*

*Pathology*	*Injuries per 1000 hours (95% CI)*	*Days (95% CI)*

**Muscle/Tendon**	***7 (3 – 11)***	-
*Muscle injury*	6 (3 – 10)	-
*Tendinopathy*	1 (0 – 2)	-
**Nervous**	***16 (10 – 22)***	***22 (21 – 23)***
*Central Nervous System*	16 (10 – 22)	22 (21 – 23)
**Bone**	***5 (1 – 8)***	***19 (6 – 32)***
*Fracture*	4 (1 – 6)	25 (21 – 29)
*Bone stress fracture*	1 (0 – 2)	-
*Bone contusion*	1 (0 – 2)	7
**Ligament/Joint Capsule**	***8 (4 – 12)***	***29 (19 – 38)***
**Superficial tissue/skin**	***1 (0 – 3)***	***14***
*Contusion (superficial)*	1 (0 – 2)	14
*Abrasion*	1 (0 – 2)	-
**Cartilage/Synovium/Bursa**	***1 (0 – 2)***	***63***
*Cartilage Injury*	1 (0 – 2)	63
Other**	***1 (0– 2)***	**-**

***TOTAL***	***38 (29 – 47)***	**20 (23 – 25)**

* Estimated severity for Time-Loss was used from data provided by the Tournament Doctors at the venue when real-time severity could not be determined.

** All Other injuries were soft tissue injuries. Due to the nature of the data collection process, we were unable to delve deeper into determining a specific diagnosis.

**Table 7:** Number of injuries and injury rates (95% CI)/1000 exposure hours for players who started the match and those who came on as substitutes in the 2024 SARU Boys’ Youth Week tournaments. Missing data = 2 cases.

	Started match		Substitution	

	Number	Incidence	Number	Incidence
**GKu16**	32	36 (23–48)	6	7 (1–12) *
**CWu18**	26	31 (19–43)	0	-

***Combined Total***	***58***	***33 (25–42)***	***6***	***4 (1–6)*** *

* Significantly lower than players who started matches

**Table 8:** Number and incidence of concussions (95% CI)/1000 player hours at the 2024 SARU Boys’ Youth Week tournaments.

Tournament	Number	Incidence	Number of matches per concussion event
**GKu16**	18	20 (11 – 29)	2
**CWu18**	8	10 (3 – 16)	3

***Combined Total***	***26***	***15 (9 – 21 )***	***2***

## References

[1] Fuller CW, Molloy MG, Bagate C (2007). Consensus statement on injury definitions and data collection procedures for studies of injuries in rugby union. Br J Sports Med.

[2] Bahr R, Clarsen B, Derman W (2020). International Olympic Committee consensus statement: methods for recording and reporting of epidemiological data on injury and illness in sport 2020 (including STROBE Extension for Sport Injury and Illness Surveillance (STROBE-SIIS)). Br J Sports Med.

[3] Fuller CW (2017). A Kinetic model describing injury-burden in team sports. Sport Med.

[4] Schenker N, Gentleman JF (2001). On judging the significance of differences by examining the overlap between confidence intervals. Am Stat.

